# Averting the Next Credibility Crisis in Psychological Science: Within-Person Methods for Personalized Diagnostics and Intervention

**DOI:** 10.17505/jpor.2021.23795

**Published:** 2022-01-07

**Authors:** Julia Moeller

**Affiliations:** Leipzig University, Germany

**Keywords:** within-person methods, theory-method gap, personalized learning, personalized medicine

## Abstract

Personalizing assessments, predictions, and treatments of individuals is currently a defining trend in psychological research and applied fields, including personalized learning, personalized medicine, and personalized advertisement. For instance, the recent pandemic has reminded parents and educators of how challenging yet crucial it is to get the right learning task to the right student at the right time. Increasingly, psychologists and social scientists are realizing that the between-person methods that we have long relied upon to describe, predict, and treat individuals may fail to live up to these tasks (e.g., Molenaar, 2004). Consequently, there is a risk of a credibility loss, possibly similar to the one seen during the replicability crisis (Ioannides, 2005), because we have only started to understand how many of the conclusions that we tend to draw based on between-person methods are based on a misunderstanding of what these methods can tell us and what they cannot. An imminent methodological revolution will likely lead to a change of even well-established psychological theories (Barbot et al., 2020). Fortunately, methodological solutions for personalized descriptions and predictions, such as many within-person analyses, are available and undergo rapid development, although they are not yet embraced in all areas of psychology, and some come with their own limitations. This article first discusses the extent of the theory-method gap, consisting of theories about within-person patterns being studied with between-person methods in psychology, and the potential loss of trust that might follow from this theory-method gap. Second, this article addresses advantages and limitations of available within-person methods. Third, this article discusses how within-person methods may help improving the individual descriptions and predictions that are needed in many applied fields that aim for tailored individual solutions, including personalized learning and personalized medicine.

Psychology aspires to understand what determines the behavior and mental states of, and differences between, *individuals*, in contrast to sociology or other social sciences, which aspire to describe and predict the behavior and functioning of *groups*. The term *individual* signals that we widely accept that two persons are not two copies of the same, but that they differ from one another in many regards. Understanding individuals, predicting their individual behavior, and finding the matching treatment for the right person at the right time is currently a defining trend in many disciplines, including the fields of personalized learning (e.g., Dumont, 2019; Bulger, 2016), personalized medicine (e.g., Senn, 2016; 2018), personalized advertisement (Zhu & Chang, 2016; Bang & Wojdynski, 2016), and many more. These goals of understanding, predicting, and treating individuals all require statistical methods that make it possible to describe how experiences develop, and how they interact within individuals (i.e., within-person methods). However, much previous research in psychology, education, medicine, and many social sciences, relies largely on methods that *do not* examine such within-person patterns (e.g., Molenaar, 2004). Consequently, it is often unknown what happens inside of an individuals’ mind; the goals of a tailored understanding, prediction and treatment for specific individuals may be difficult to reach, and many psychological studies attempting to study individuals may fail to actually do so.

A methodological revolution appears imminent, indicated for instance by several “manifestos” in which eminent experts have demanded “bringing the person back into psychological science” by using within-person methods (Molenaar, 2004, p. 201; Barbot et al., 2020; Renner et al., 2020). Various within-person methods have been proposed as solutions for better descriptions of individuals (e.g., Beltz et al., 2016; Völkle et al., 2014; von Eye, 2003), but neither do they solve all the known methodological problems of describing individuals, nor have they been sufficiently applied in studies on psychological theories, in personalized learning, or other disciplines aiming for personalization, such as personalized medicine.

This article gives an overview of some crucial limitations in the commonly used between-person methods in order to explain why and in what regards there may be an imminent risk for the emergence of a new credibility crisis in psychology (called a validity crisis by Lundh, 2019), due to systematic discrepancies between the conclusions about groups that our methods allow us to draw, and the conclusions about individuals that we tend to draw based on these methods. The article then proceeds to give an overview of some available within-person methods that solve some of the limitations of the between-person methods. After that, the article discusses why the limitations of between-person methods, the potential of within-person methods, and the demand for personalization in many applied fields together are likely to lead to paradigmatic changes in psychological research, including methods, theories, and applications. The article closes with the conclusion that a pro-active reckoning about the theory-method gap in psychology is needed to avert a new credibility crisis (or validity crisis; see Lundh, 2019), and proposes a research program for this proactive self-improvement of psychological science.

## 1. A Theory-Method Gap in Psychology: Limitations of Common Between-Person Methods

Although psychology aims to describe and predict how individuals feel, think, and behave, many of the analytical and diagnostical methods used to study such questions about individuals focus mostly on group-based statistics, such as mean-score differences between groups, or group-based correlation or regression coefficients. This section summarizes reasons why and in what cases such group-based between-person statistics may fail to describe some, or even all, of the individuals in the studied sample. The leading theme in this section is the concern about a systematic theory-method gap in many areas of psychology, in the sense that we often study theories and hypotheses about within-person patterns or processes with between-person methods that are unsuited to tell us anything about the within-person pattern or processes of interest.

### 1.1. Between-Person Methods Do Not Sufficiently Describe Change Within Individuals Due to Simpson’s Paradox

One reason why group-based statistics may fail to describe many individuals in the studied sample is the possible presence of Simpson’s (1951) paradox. Understanding processes of change in a person requires multiple assessments of the same person over time, in combination with methods to analyze the within-person trajectories over time (see Reitzle & Dietrich, 2019; Curran et al., 2014). Such methods can help teasing apart the aspects that change and fluctuate (states) from those that distinguish one person from the other and remain stable in a person across multiple measurements (traits).

For instance, teachers want to know what they have to do in a teaching situation to change a learner’s interest or knowledge; doctors want to know how to treat a patient in a way that best changes their health for the better; advertisers want to know which malleable attitudes and behaviors they can affect with which advertisement stimuli; and all these protagonists may want to know how stable person characteristics should inform decisions about situation-specific and person-specific assessments and treatments. Different individuals may change in different ways, and to understand such between-person differences in within-person change processes it is helpful to first examine for each person how their experiences change over time, and then examine whether groups of individuals with similar change patterns can be identified (e.g., Beck & Jackson, 2020; Beltz et al., 2016).

If between-person methods are applied without the previous step of identifying the trajectories within individuals first, then misinterpretations can arise. For instance, imagine data consisting of multiple individuals with multiple measurement time points per person that are used to study whether a construct changed over time. A typical between-person approach would either calculate a group-based average of the variable for each time point (averaging the variable across individuals within each time point) and then examine changes in these group averages from one time point to the next (called mean-level stability), or examine between-person correlations of the variable from one time point to the next (called rank-order stability; for studies using both techniques, see e.g., Mõttus et al., 2012; Specht et al., 2011). A problem with both approaches is that the individual trajectories of the persons in that sample can look very different from, and therefore *cannot be deduced from such group-based statistics* (e.g., Reitzle & Dietrich, 2019). For instance, a construct can appear to increase over time according to between-person methods, while in fact it tends to decrease within each person, or vice versa, which is the longitudinal version of the problem known as Simpson’s (1951) paradox or as a lack of ergodicity (e.g., Molenaar, 2004; for its role in longitudinal studies, see Kievit et al., 2011; 2013; Yarnold, 2013). Thus, neither mean-level stability nor rank-order stability estimated with between-person methods tell us whether, how much, and in which directions, a construct changed within a person, or which trajectories were observed in which – and how many – individuals.

### 1.2. Simpson’s Paradox May Also Obfuscate the Structure of One Construct, or the Relationships Among Multiple Constructs

The problem that within-person patterns occasionally differ from group-level trends (also described as Simpson’s paradox^1^ or lack of ergodicity^2^) does not only mean that within-person trajectories over time in one variable cannot be deduced from between-person analyses of mean-level or rank-order stability. *The same problem also applies to analyses of the structure of a construct, or the relations among more than two psychological constructs*. It implies that interrelations (e.g., correlations, regression coefficients) among multiple construct indicators or constructs often look vastly different if examined with within-person methods than with between-person methods (e.g., Molenaar, 2004). This means, for instance, that the factor structure of one construct, the results of a factor analysis, or the relations among more than two variables in structural equation models can look different in between-person analyses than in within-person analyses. It means that a between-person analysis can propose a structure of interrelations among multiple variables that fails to describe how the very same variables relate to each other within individuals. Some between-person findings may properly describe the experiences of at least a subgroup of individuals, others may fail to describe any individual’s experiences at all.

Detecting such within-versus between-person differences in the structure or interrelations of constructs typically requires multiple measurement time points per person (i.e., intensive longitudinal data), so that within-person correlations or regression coefficients can be examined across these multiple time points (e.g., Beck & Jackson, 2021; Cattell, 1946). Therefore, many of the studies addressing either this problem or the solutions to it can be found in research using such intensive longitudinal data, many of which conclude that interrelations among psychological constructs are best described with combinations of intra- and between-person methods (e.g., Brose et al., 2020; Völkle, et al., 2014; Murayama et al., 2017).

The lack of ergodicity or Simpson’s paradox is currently the most frequently mentioned argument for within-person methods and a leading argument in the various manifestos calling for within-person methods (e.g., Barbot et al., 2020; Molenaar, 2004; Renner et al., 2020).

### 1.3. Between-Person Approaches Report One-Size-Fits-All Effects, But Heterogeneity Can Hide Behind Such Overall Trends

**Figure 1:**
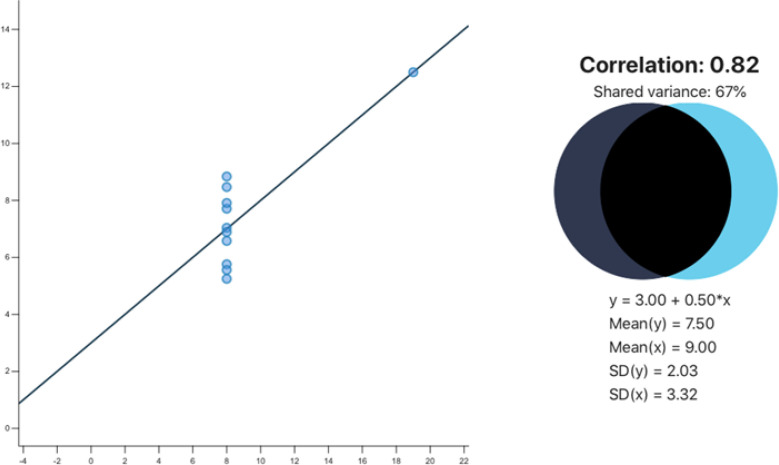
Anscombe’s fourth quadrant, data source: Anscombe (1973). The figure was created using the interactive correlation simulation provided by Magnusson (2021).

Between-person approaches typically report one result for the entire sample (e.g., one correlation, or one regression coefficient), which follows a one-size-fits-all logic. However, it is known that *heterogeneity can hide behind such overall trends*, and that, for instance, individuals with patterns opposing the overall trend may be overlooked (see e.g., Anscombe, 1973; Matejka & Fitzmaurice, 2017). This problem has been long known and is yet often ignored (e.g., Asendorpf, 1993; Asendorpf, 2000; Kuhl, 1977; Lewin, 1930; Wottawa, 1981). It implies that *without examining scatter plots and bivariate distributions more in detail, we do not know how many individuals in the sample behave in the way a sample’s inter-individual correlation or regression coefficient suggests*. A sample’s correlation or regression coefficient may fail to describe the variable patterns experienced by some or even all individuals in the sample: As Anscombe’s (1973) fourth quadrant (see Figure 1 below) shows, it is even possible to find a strong correlation or regression coefficient in a sample where there is no systematic relation between the X and the Y variable at all.

**Figure 2:**
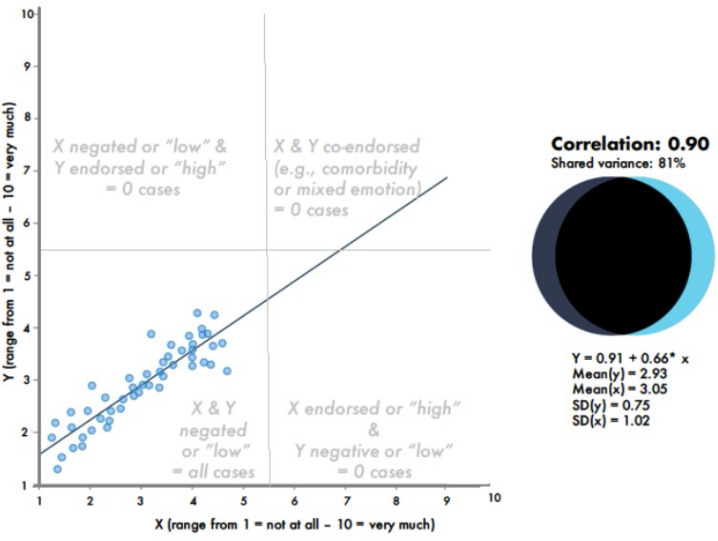
A case of a strong correlation (r = .90), despite lacking endorsement in both variables (assuming for the sake of the argument that the midpoint of either response scale – here: score 5 – represents the distinction between item endorsement and item rejection). The figure was created using the interactive correlation simulation provided by Magnusson (2021), with the dotted lines, grey comments and axis labels added by this article’s author.

Vice versa, as Matejka and Fitzmaurice (2017) show, it is possible to find a zero correlation in a sample where about half of the individuals show patterns in line with a perfectly negative correlation between X and Y, while the other half of the individuals show patterns in line with a perfectly positive correlation between X and Y (see the dots in the scatter plot in Matejka and Fitzmaurice’s [2017] Figure 2, where roughly half of the dots/individuals lie along a diagonal from top left to bottom right, representing a negative correlation, whereas the other half of the observations lie along a diagonal from the bottom left to the top right, representing a positive correlation, while the overall correlation for that sample is around zero). For these reasons, inter-individual correlations or regression coefficients cannot automatically be assumed to describe the patterns of variables within individuals. Asendorpf (2000) calls this fallacy of misinterpreting an inter-individual finding as if it applied to individuals the “idiographization of a nomothetic finding” and proposes to report the percentage of individuals who show patterns in line with the sample-level nomothetic finding. So far however, misinterpretations of correlations or regression coefficients as within-person patterns are and have been persistent among even the highest educated psychologists (Valsiner, 1986).

In particular, any research attempting to understand heterogeneity and the aspects in which individuals may deviate from general trends requires methods that are able to discover such heterogeneity hiding behind between-person overall correlations or regression coefficients. This is particularly relevant for any research attempting to personalize diagnostics and treatment, such as personalized learning, inclusive education, personalized medicine, or personalized advertisement.

### 1.4. Covariance Is Not Co-Endorsement, But Is Often Mistaken for Such

Another example of frequently misinterpreted between-person coefficients is the interpretation of correlation or regression coefficients as if they revealed whether one variable Y is high if the other variable X is high (positive correlation) or low (negative correlation). Many researchers conclude that a negative correlation implies that individuals with “high” levels in one variable show “low” levels in another variable; and that a positive correlation implies that individuals tend to show similar levels in both variables (i.e., high levels in both variables, or moderate levels in both, or low levels in both). However, this frequent interpretation ignores that *between-person correlations or regression coefficients say nothing about whether an individual endorses two items, or denies two items, or has a high score in reference to an absolute response scale* (see e.g., Asendorpf, 2000; Moeller, 2018a). Correlations and regression coefficients are independent of whether any item is *endorsed* alone or together with another item by anyone in the sample. Covariance is not the same as co-endorsement (see Figure 2)

Thus, any research trying to find out whether two items are endorsed together, such as research on comorbidity (asking which pathological symptoms are experienced together) or mixed emotions (asking which emotions are experienced together), needs methods capable of describing *within-person co-endorsements* of sets of items (see e.g., Moeller et al., 2018a). For a summary of the discussions about reasons why correlation coefficients are not well suited to study mixed emotions, please see Larsen and McGraw (2014).

Statements about co-variance do not carry information about the *absolute scores* in variables, only information about the *relative rank* of individuals in reference to the distribution of all individuals. For instance, two variables can be positively correlated, seemingly suggesting that if X is high, Y is also high, and yet the inter-individual average of one of these variables can be much higher (e.g., variable X can have a mean of seven on a scale from one to ten) than the other (e.g. variable Y can have a mean of three on a scale from one to ten), and this relation of absolute scores of X > Y can be true for all individuals in that sample. In this example, it could be argued that variable Y is never “high” in terms of an item endorsement, despite of the positive correlation between both variables. For example, the desirable (harmonious) form of passion and the undesirable (obsessive) form of passion described in Appendix B are positively correlated with each other, and nevertheless obsessive passion is typically denied by most individuals and in most cases lower than the same individual’s harmonious passion (Moeller et al., 2015). Thus, it is important to keep in mind that covariance refers to individuals’ *relative* ranks in the distribution of individuals, and that this can be a vastly different from what many people understand when they hear that a variable is “high” or “low”, by which many researchers mean the absolute position on a response scale (e.g., score 3 of 10 versus score 7 of 10).

The problem that we tend to mix up a person’s rank in relation to others with a person’s score on a bound response scale extends beyond the issues of interpreting correlation or regression coefficients. The same principle is responsible for the problem that we often misinterpret above-average *z*-standardized scores as if they represented item endorsements, when in fact they may represent an item rejection. For instance, due to the relatively low mean score (or high item difficulty) of obsessive passion, an above-average *z*-score of *z_obsessivePassion_* > 0 often represents individuals who rejected the items of the obsessive passion scale, but due to mix-ups of relative rank and absolute positions on response scales, such individuals have been interpreted as obsessively passionate individuals (for a critique and summary, see Moeller et al., 2015; for further discussions of this problem, see also Moeller, 2015; 2020). Thus, while inter-individual *z*-scores only hold information about a relative rank of a person *in relation to other individuals* (between-person comparison), they are often interpreted as if they reflected information about item affirmation of individuals. If ranks in inter-individual comparisons are misinterpreted as information about item endorsement or as information about “high” or “low” scores in terms of a response scale, the between-person reference is confused with information that could be interpreted intra-individually. Consequently, misinterpreting relative ranks as information about positions of responses on an absolute response scale can lead to several fallacies:

*Fallacy 1*: The first fallacy is to confuse co-variance with co-endorsement by mistaking a positive correlation as evidence for variable Y being “high” if variable X is “high” or by interpreting a negative correlation as evidence for variable Y being “low” if variable X is “high”. A more precise interpretation would be to interpret a positive correlation in the way that individuals with *higher* scores in variable X (*compared to other individuals in the same sample*) tend to have *higher* scores in Y (*compared to other individuals in the same sample*), but please also consider the heterogeneity and ergodicity problems noted above.

*Fallacy 2*: The second fallacy would be to interpret relations between two or more ranks in inter-individual comparisons as if they described within-person relations between responses on absolute response scales. If two variables are *z*-standardized using the inter-individual mean score and standard deviation, then an individual’s relation between the *z*-scores of variables X and Y does not translate into this person’s raw scores on the same unstandardized variables. For a given person with *z_VariableX_ > z_VariableY_*, the raw scores of X and Y may come in any one of the constellations *rawVariableX < rawVariableY*, *rawVariableX > rawVariableY*, or *rawVariableX = rawVariableY*. As an example of research committing this fallacy, many studies interpret above-average *z*-scores as if they represented item endorsements when in fact they may represent item rejections. See for instance the research on flow where above-average *z*-scores of situational challenge are interpreted as “high challenge” despite the fact that many above-average ratings of situational challenge represent item rejections due to the rather low mean score (e.g., Schneider et al., 2016). Another example is the research on passion where above-average *z*-scores on obsessive passion are interpreted as “high obsessive passion” despite the fact that many above-average ratings of obsessive passion represent item rejections due to the low mean score (for an overview, see Moeller et al., 2015).

**Figure 3:**
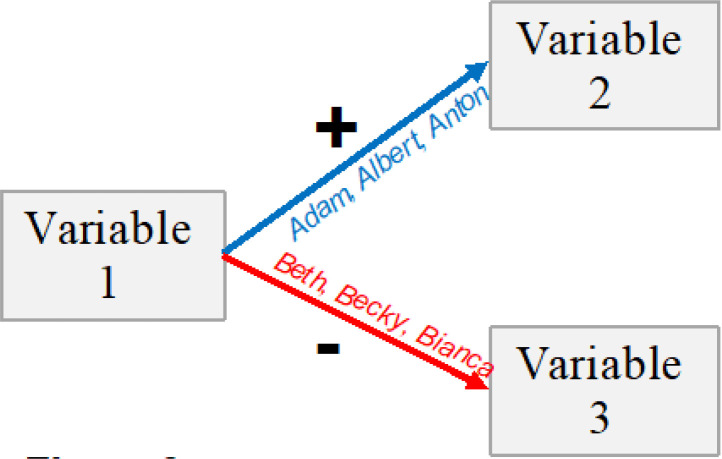
Different people can account for the covariance represented by different paths in between-person path analyses and between-person structural equation models

As a further example of the same Fallacy 2, when examining Figure 3 we may be tempted to believe that the relations between the regression coefficients of the blue paths to the red paths could be interpreted as relations in the *scores* of variables 2 to variable 3 when controlling for variable 1. That is, we might be tempted to believe that when controlling for variable 1 in Figure 3, variable 2 should be higher than variable 3, because variable 1 is positively correlated with variable 2 but negatively with variable 3. However, again, the ratios of the two between-person rankings, represented by the correlation or regression coefficients, *do not translate* into within-person ratios of the scores of any of the variables in terms of absolute response scales. Even though variable 1 is positively correlated with variable 2 and negatively with variable 3, variable 3 can still be higher than variable 2 in every individual in the sample. It is therefore important to keep in mind that between-person ranks do not translate into within-person positions of scores on response scales, nor can ratios among between-person ranks be interpreted as if they reflected within-person patterns in terms of response scale positions or item endorsements.

### 1.5. Between-Person Structural Equation Models Are Often Interpreted as If They Reveal Within-Person Relationships Among Variables, Which They Do Not

Many studies examine between-person relations (including causal relations) among sets of *more than two* psychological variables by examining the between-person co-variance among these variables with path analyses or other structural equation models (SEM; e.g., Ullman & Bentler, 2003; see Figure 3).

Typically, many researchers assume that the various paths in the model describe experiences of the same person. Therefore, we can expect to see a model such as the one depicted in Figure 3 being interpreted as if it indicated that people with relatively high scores in variable 1 tend to have relatively high scores in variable 2, but low scores in variable 3. However, it has been pointed out that such a commonly seen interpretation is based on a misunderstanding. *A path analysis like the one depicted in* *Figure 3* *does not imply that any individual “walks” all the paths* (Reitzle, 2013).

Instead, it is possible for one group of individuals (e.g., Adam, Albert, and Anton) to account for the positive covariance depicted in one path (such as the blue path in Figure 3, or the positive covariance between math ability and math self-concept depicted in Figure 5), while an entirely different group (e.g., Beth, Becky, and Bianca) may account for the covariance depicted in another path (such as the red path in Figure 3, or the negative covariance between the math performance and the English ability self-concept depicted in Figure 5). Thus, path models do not necessarily describe the patterns among the studied variables *within* a person, which has been demonstrated both theoretically and empirically (Reitzle, 2013).

**Figure 4:**
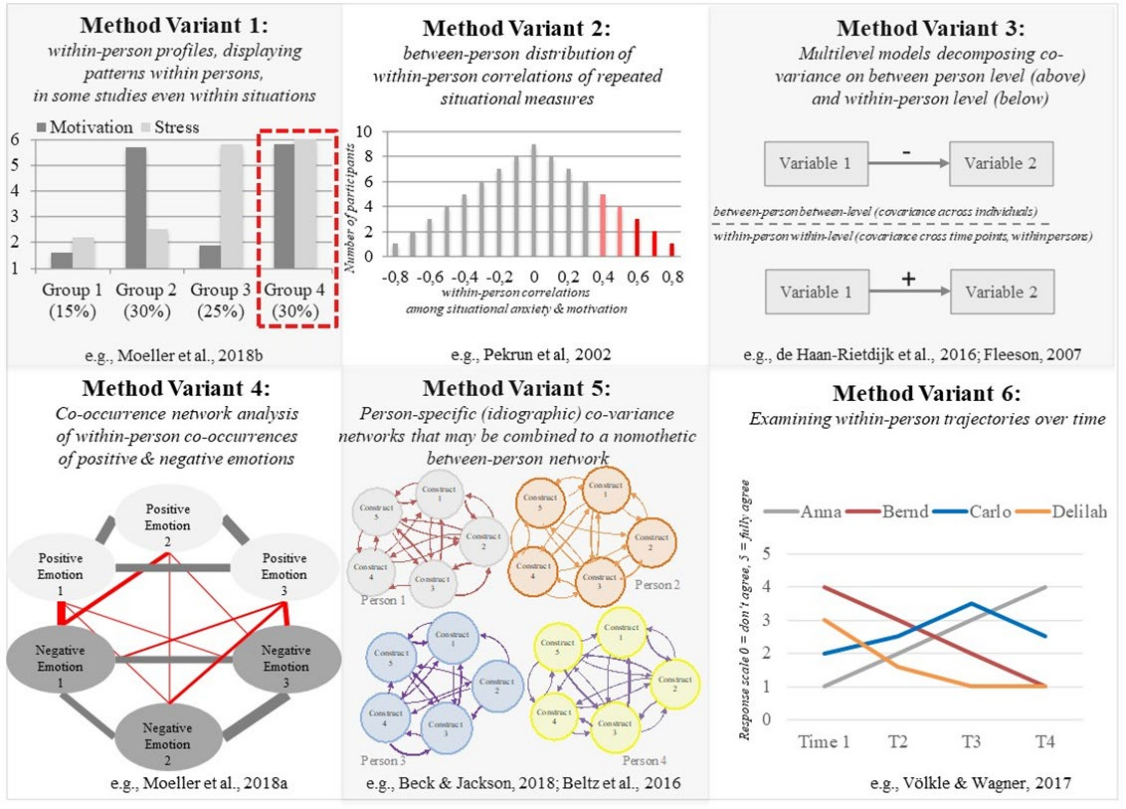
Variants of within-person methods

This has tremendous implications for the debate about the use of cross-lagged panel models and mediation analyses in the study of causality (Hamaker et al., 2015; Rogosa, 1980). That different sets of individuals can drive the covariance behind different paths means that, for mediation models the path from a predictor to a mediator may be driven by different individuals than the paths from the mediator to the outcome, or the direct path from the predictor to the outcome. For cross-lagged panel models, it means that *we cannot interpret paths as causal relations occurring within individuals*, which comes in addition to all the other limitations of a causal interpretation of cross-lagged panel models (e.g., Hamaker et al., 2015; Rogosa, 1980).

Causality in terms of psychological processes often refers to intra-individual relations among variables (e.g., eating breakfast causing a person to feel less hungry in the next two hours; having a conflict with a parent causing a teenager to be grumpy afterwards, see Lichtwarck-Aschoff et al., 2009). Inter-individual covariance-based analyses (such as CFA/ SEM, path analyses, and cross-lagged panel models), however, *do not examine such intra-individual causal relations among constructs*.

It should be noted that some of these above-mentioned limitations of between-person methods have been known and discussed by some researchers for a long time (for an overview, see e.g., Asendorpf, 2000). Some are more frequently discussed (such as points 1.1; 1.2, and 1.3 above), whereas others are less widely considered (points 1.4 and 1.5 above). In the light of these limitations that between-person methods face in describing individuals, various within-person methods have been lauded as possible solutions. The next section therefore discusses the within-person analyses that are available, which problems they solve, and what limitations they have.

## 2. Within-Person Methods Help Fill Some of the Gaps Left by Between-Person Methods

Within-person analytical methods examine how variables change within a person over time, or how various variables relate to each other within a person at one time point. This section particularly addresses and explains the six variants of within-person analysis displayed in Figure 4.

### 2.1. Analysis of Within-Person Profiles (e.g., Cluster Analysis, Latent Profile Analysis, Latent Class Analysis; Figure 4, Example 1)

Cluster analyses or the latent variants thereof (latent profile analyses) can reveal within-person profiles of two or more variables and indicate how many individuals experience which intra-individual profile pattern. For example, latent profile analyses revealed that despite a negative correlation between burnout and engagement (a form of motivation), about 20-30% of all students and employees in various samples experienced high levels of both engagement and burnout (Moeller et al., 2018b; Salmela-Aro et al., 2016; Tuominen-Soini & Salmela-Aro, 2014). Thus, this method can be used to report the percentage of individuals who do, or do not, show patterns in line with sample-level correlation or regression coefficients or path models, as proposed for instance by Asendorpf (2000). The above-mentioned group of engaged but burned-out individuals would be overlooked if only the significantly negative between-person correlation of these variables were examined.

Profile analyses can also be used to identify in-the-moment profiles For example, they can show one profile (e.g., high scores on motivation, stress, and anxiety) occurring in situation 1; another profile (e.g., a high score on motivation co-occurring with low scores on anxiety and stress) occurring in situation 2; and a third profile (e.g., high motivation combined with high stress but low anxiety) occurring in situation 3. In this case, one person can experience multiple profiles, one at a time, which may change from one moment to the next (e.g., Bergman et al., 2012; Dietrich et al., 2019).

### 2.2. Within-Person Correlations Between Two Variables That Are Measured Repeatedly in Each Person in Multiple Situations (Figure 4, Example 2)

To find out how two variables are related to each other within individuals, it can be useful to examine the within-person correlation or regression coefficient among two (or more) repeatedly measured variables. This method is typically used if a large number of longitudinal measurement time points (approximately *N* > 30) are available for each person, which is often the case with intensive longitudinal data (e.g., Moeller et al., 2015; Pekrun et al., 2002). One variant of this approach has been used by Pekrun et al., (2002) and Moeller et al., (2015), who plotted the inter-individual distributions of such within-person correlations among repeatedly measured variables (in these studies: situational anxiety and situational measures of motivation and various positive emotions; see Figure 4, Example 2).

In a next step, the inter-individual distribution of the intra-individual correlations can be examined to address heterogeneity (e.g., the within-person correlation between anxiety and motivation being positive for some but negative for other individuals, see Pekrun et al., 2002) or to address possible moderators (e.g., the within-person correlation between situational anxiety and negative emotions being stronger for female than for male students, see Moeller, Salmela-Aro, et al., 2015).

### 2.3. Multilevel Models Decomposing Within- and Between-Person Variance (Figure 4, Example 3)

As an extension of or as a basis for the previously described within-person correlations (see Figure 4, Example 2 above), multilevel modeling can be used to decompose the variance of a repeatedly measured variable into the variance between multiple time points within each person (“within-level”) and the variance between individuals in regard to each person’s average across time (“between-level”). For examples, see Brose et al. (2020); Völkle et al. (2014); Moeller et al. (2017; 2020c), and Figure 4, Example 3. The advantage of this method is that it can reveal discrepancies between the covariance patterns of the within-person versus the between-person level (i.e., the compositional effect, see Raudenbush & Bryk, 2002), which is why it is currently being lauded as a solution to the problems of Simpson’s paradox or lack of ergodicity in longitudinal data (described in section 1.1 above; see e.g., Brose et al., 2020; Völkle et al., 2014).^3^ The multilevel variance decomposition can be extended to include structural equation models, including path analyses or confirmatory factor analyses, to model covariance patterns among larger sets of variables that may differ between the within-person and the between-person levels.

### 2.4. Co-Occurrence or Co-Endorsement Network Analysis (Figure 4, Example 4)

To account for the problem that some research questions require answers about within-person co-endorsement patterns that analyses of covariance cannot provide (see section 1.4 above), the co-variance network analysis examines how often two variables are affirmed (i.e., endorsed) together, or mentioned together, by the same person (for a description of the method, see Moeller et al., 2018a). The co-endorsement analysis is particularly relevant for all studies that focus on joint experiences, such as research on co-morbidity (asking which clinical symptoms are experienced together) and research on mixed emotions (asking which emotions of mixed valence are experienced together). In the co-endorsement network, the psychological variables (e.g., emotions) are represented by bubbles (called *nodes*), and every time a person affirms a pair of these variables together, the line (called *edge*) between these nodes becomes thicker. For example, the thin red paths in Example 4 in Figure 4 indicate that positive and negative emotions are affirmed together occasionally, but not as often as emotions of the same valence are affirmed together (thicker grey lines).

### 2.5. Network Models Examining Estimates of Co-Variance (Figure 4, Example 5)

As an extension of the analysis of within-person covariance (correlations or regression coefficients; see method examples 2 and 3 in Figure 4), covariance-based network analyses can be used to examine within-person correlations or regression coefficients among multiple variables. There are various unique advantages and insights to such covariance-based within-person networks. For instance, some variants of co-variance-based networks distinguish between person-specific networks of individual persons (called idio-graphic) and general networks representing between-person trends (called nomothetic). Employing the variance decomposition described above in point 2.3 (multilevel models), this approach acknowledges that each individual may show unique within-person patterns of how the studied variables co-vary over time.

In addition to these person-specific networks, of which there are as many as there are individuals in the sample, one separate between-person (nomothetic) network can be estimated to display those aspects of these person-specific networks that were empirically found to generalize reliably across individuals. A statistical approach of integrating person-specific (idiographic) and general between-person (nomothetic) approaches is called GIMME (group iterative multiple model estimation; Beck & Jackson, 2020; Beltz & Gates, 2017; Gates & Molenaar, 2012; Beltz et al., 2016; Wright et al., 2019). The GIMME method can also identify subgroups of individuals sharing similar sets of associations among the studied variables to account for heterogeneity between individuals in regard to their within-person correlational associations (see e.g., Gates et al., 2017).

### 2.6. Analysis Of Within-Person Trajectories Over Time (Figure 4, Example 6)

Understanding the changes that people go through requires an understanding of how things change within individuals. To study such within-person trajectories, we need methods that estimate person-specific intercepts and slopes, such as the analysis of within-person trajectories in within-person growth curve models (e.g., Pasyugina et al., 2015). This principle of estimating person-specific trajectories is often combined with the multilevel variance decomposition into within-person and the between-person levels (see Figure 4, Example 3 above) in so-called random coefficient regression models (e.g., for a description of the method, see Cohen et al., 2003). To find inter-individual clusters of similar within-person trajectories, growth curves can be combined with clustering approaches, by clustering similar individual trajectories into homogeneous groups and counting the distinct groups’ frequencies (e.g., Muthén & Muthén, 2000), the latter of which is a way to report the percentage of individuals who show patterns in line with sample-level results, as proposed by Asendorpf (2000).

### 2.7. Which Gaps Left by Between-Person Methods Are Filled by Which Within-Person Methods?

Within-person methods are expected to be game-changing when it comes to describing and predicting individuals. The reasons for this expectation are mainly that within-person methods provide unique insights that go beyond those provided by the more common between-person approaches, whereas previous research in psychology and many other social sciences has largely relied on the limited between-person methods so far. Table 1 gives an overview of the above-described within-person methods and which of these are expected to solve which of the described problem in describing individuals.

**Table 1:** Problems of between-person methods (rows) and within-person methods that may help to solve them (columns). Numbers 1.1 – 2.6 refer to the matching numbered sections in this article.

*Within-person methods described above*	2.1. Within-person profiles and scatter plots^1^	2.2. Between-person distributions of within-person correlations^2^	2.3. Multilevel corre-lation or regression; situations nested in individuals^3^	2.4. Network analysis show-ing within-person co-endorsements^4^	2.5. Combinations of person-specific/within-person and between-person co-variance-based networks^5^	2.6. Analyses of within-person trajectories (e.g., within-person slopes & intercepts)^6^
*Limitations of between-person methods described above*						
1.1. Understanding change requires analyzing within-person trajectories. Between-person methods may misin-terpret trajectories^7^	*If scatter plots show repeated measures separately for distinct individuals*.				*If networks examine mo-ment-to-moment autoregres-sive paths within persons*	**Problem 1.1 can be solved by solution 2.6**
1.2. Processes and structures of psychological constructs often differ in within-person versus between-person analyses (lack of ergodicity)^8^	*If scatter plots show repeated measures separately for distinct individuals*.	**Problem 1.2 can be solved by solution 2.2**	**Problem 1.2 can be solved by solution 2.3**	*If only single edges are interpreted or if idiographic and nomothetic networks are distinguished*	**Problem 1.2 can be solved by solution 2.5**	*If between-person differ-ences in regard to within-person trajectories are addressed*.
1.3. Heterogeneity & unexpected pat-terns hiding behind a between-person coefficient^9^	**Problem 1.3 can be solved by solution 2.1**	*Does reveal differences between individuals but does not solve the problems described by* *Anscombe (1973)*	*Does reveal differ-ences between indi-viduals but does not solve the problems described by* *Anscombe (1973)*	*Partially, can be used to distinguish covariance from co-endorsement, but does not represent entire bivariate distribution*	*Does reveal differences be-tween individuals but does not solve the problems de-scribed by* *Anscombe (1973)*	*Does reveal differences between individuals but does not solve the problems described by* *Anscombe (1973)*
1.4. Co-variance mixed up with co-endorsement^10^	**Problem 1.4 can be solved by solution 2.1**			**Problem 1.4 can be solved by solution 2.4**		
1.5. Different people ‘walking’ different paths in path models^11^	*Only profile analysis, not scatter plots*			*Adaptation needed to dis-tinguish idiographic and nomothetic networks*	*Solved only in idiographic networks*	*If all trajectories in the model were examined withinsame person*.

*Note*. ^a^ = Moeller et al., 2018b; ^2^ = Pekrun et al., 2002; Moeller et al., 2015; ^3^ = Brose et al., 2020; Dietrich et al., 2017; Völkle et al., 2014; ^4^ = Moeller et al., 2018a; ^5^ = e.g., Beck & Jackson, 2020; Gates & Molenaar, 2012; Beltz et al., 2016; Wright et al., 2019; ^6^ = Moeller et al., in press; ^7^ = e.g., Reitzle & Dietrich, 2019; ^8^ = e.g., Molenaar, 2004; Yarnold, 2013; Kievit et al., 2013; Kievit et al., 2011; Vansteenlandt et al., 2015; Völkle et al., 2014; ^9^ = discussed by Anscombe, 1973; Matejka, & Fitzmaurice, 2017; Asendorpf, 1993; 2000; ^10^ = discussed in Moeller et al., 2018a; ^11^ = discussed in Reitzle, 2013. In the cells, complete solutions to problems are marked bold, partial solutions are marked in italics.

*Examining intra-individual scatter plots of bivariate distributions* is a powerful and much under-used instrument. It can help to detect Simpson’s paradox (see for example the group-wise scatter plots by Kievit et al., 2013), which helps solving the problems 1.1 and 1.2, both of which refer to lack of ergodicity in longitudinal data. Scatter plots also help detecting unexpected bivariate distributions that deviate from the pattern that a between-person regression or correlation coefficient suggests, which solves the problem described by Anscombe (1973) and Matejka and Fitzmaurice (2017). Scatter plots also reveal differences between covariance and co-occurrence discussed in section 1.4. As a limitation, bivariate scatter plots do not reveal whether the same individuals account for multiple paths in path analyses or other structural equation models (the problem described in section 1.5 and by Reitzle, 2013).

Analyses of within-person profiles (or clusters of individuals with distinct profiles) can be used in similar ways. They can be used to examine each person’s intra-individual trajectory (see Figure 4, Method Variant 6 and the problem described in section 1.1) or each person’s intra-individual profile of multiple variables (Figure 4, Method Variant 1 and the problem described in section 1.2). Also, the analyses of within-person profiles may be somewhat helpful in discovering some of the heterogeneity that can hide behind overall trends (see the problem described in section 1.3 and e.g., Moeller et al., 2018b) and they can be useful to address the distinction between covariance and co-occurrence (see Moeller et al., 2018b). In contrast to bivariate scatter plots, the analyses of within-person profiles can show a person’s score in more than two variables, which can be a helpful tool in finding out “who walks which path” in terms of the problem addressed in section 1.5 and by Reitzle (2013).

*Plotting the between-person distribution of within-person correlations* described by Pekrun et al., (2002) and Moeller et al. (2015) can be used to examine between-person differences and heterogeneity in regard to within-person covariance (see section 1.2). However, the method does not fully solve the problem of unexpected bivariate patterns hiding behind correlation or regression coefficients, described by Anscombe (1973) and Matejka and Fitzmaurice (2017) and in section 1.3.

Multilevel correlation or regression analyses decomposing variance into Level 1 (variance within individuals and across situations) and Level 2 (variance between individuals) have been used as solutions to the problem of lacking ergodicity (different coefficients within versus between individuals; see section 1.2 and Brose et al., 2020; Völkle et al, 2014).^4^ Such decomposition of (co-)variance into within- and between-person sources can help to reveal heterogeneity between individuals in regard to within-person methods, but it does not solve the problem that unexpected bivariate patterns can hide behind correlation or regression coefficients (as discussed in Anscombe, 1973).

*Co-endorsement network analyses* (for an introduction, see Moeller et al., 2018a) could help to address aspects of possible discrepancies in within-versus between-person structure of variables (the problem described in section 1.2). One edge (line) in this network by definition shows the sample’s frequency of a given within-person co-endorsement of two variables and thus combines a within-person statistic (co-endorsement: yes or no) with a sample-level aggregate (relative frequency of that co-endorsement). However, the overall network pattern cannot be interpreted as a within-person pattern, because it is not guaranteed that the same individuals account for multiple edges (lines). To change that and to more precisely address the possible discrepancy between within-person versus between-person structures of variables, the co-occurrence network analysis needs to be adapted to distinguish between person-specific (idiographic) networks and sample-level networks, as it has been proposed for covariance-based networks (e.g., Beck & Jackson, 2020; Beltz & Gates, 2017; Gates & Molenaar, 2012; Beltz et al., 2016; Wright et al., 2019).

Co-endorsement network analysis do reveal the possible existence of co-endorsement patterns that defy the interpretations suggested by a correlation or regression coefficient (see Moeller et al., 2018a), which is arguably a partial solution to the problem of possible unexpected bivariate distributions hiding behind such covariance coefficients (the problem described in section 1.3). However, the co-endorsement network analysis does not examine the entire range of the bivariate distribution, and instead focuses on the upper right quadrant^5^ of a scatter plot, as Figure 2 illustrates. The method could be adapted to also examine the other three quadrants pattern of (1) joint item rejection, (2) endorsed X-variable with rejected Y-variable and (3) rejected X-variable with endorsed Y-variable.

The main purpose of the co-endorsement network analysis is to reveal possible discrepancies between the interpretations following from covariance versus co-endorsement analyses (the problem discussed in section 1.4). As with the previously discussed problem, we need to keep in mind that covariance examines the entire bivariate distribution, whereas the co-endorsement (network) analysis focuses on the upper right corner of a scatter plot (see Figure 2). This is practical for some research areas (e.g., the research on mixed emotions and comorbidity), but it may be impractical for others. This limitation can be removed by adapting the item dichotomization for the method so that all other constellations of item endorsements and rejections can be examined (see above). Some researchers will state the often-heard problem that dichotomizing metric variables leads to a loss of information, but in this case, it also leads to an increase in information – about item endorsement – that is lost if the metric variables are correlated.

The problem that multiple paths are not necessarily driven by the same individuals (described in section 1.5 and Reitzle, 2013) cannot be solved with the co-endorsement network analysis, unless it is adapted to distinguish between person-specific (idiographic) networks and sample-level (nomothetic) networks, as it has been proposed for covariance-based networks (e.g., Beck & Jackson, 2020; Beltz & Gates, 2017; Beltz et al., 2016; Wright et al., 2019).

*The combination of person-specific, idiographic networks examining covariance among a set of variables and between-person, nomothetic versions thereof*, as described for instance by Beck & Jackson (2020) and Beltz et al. (2016) can be used to describe within-person trajectories and thus provide a solution to Simpson’s paradox in longitudinal studies (the problem described in section 1.1), but only under the condition that the network examines such within-person trajectories from one measurement time point to the next, such as moment-to-moment autoregressive paths in idiographic networks (for examples, see Beck & Jackson, 2020; Chaku et al., 2021). The method does provide a full solution to the possible discrepancy between the within-versus between-person structure of constructs (the problem described in section 1.2), as this is the main purpose for which this method was developed. The method does reveal differences between individuals with regard to their within-person covariance patterns, but it does not solve the problem of possibly unexpected bivariate distributions that can hide behind coefficients based on covariance (the problem discussed by Anscombe, 1973 and in section 1.4). The idiographic networks included in this method also make sure that the multiple paths in the network can be interpreted as describing the same person (solving the problem described in section 1.5 and by Reitzle, 2013).

Finally, the analyses of within-person trajectories (e.g., within-person slopes & intercepts) does solve the problem of Simpson’s paradox in longitudinal data (described in section 1.1). The method can also be used to address possible between-person differences with regard to within-person trajectories (the problem discussed in section 1.2), if the between-person range and distribution of the individual within-person intercepts, slopes, and curves are addressed (which is the case in some studies and applications of this method). Thus, the method can be used to address heterogeneity between individuals with regard to within-person trajectories, but it cannot solve the problem that unexpected bivariate distributions can hide behind coefficients based on covariance (the problem discussed by Anscombe, 1973 and in section 1.4). Examining within-person trajectories is one way of describing the path that a person “walks”, which is arguably one solution to the problem discussed in section 1.5 and by Reitzle (2013).

Importantly, the within-person methods described in this article do not solve all of the problems described in the context of between-person methods. A detailed discussion of further limitations of the within-person methods addressed in this article can be found in Appendix A.

## 3. Personalized Descriptions and Predictions Require Person-Oriented Methods

Due to the fact that previous research in psychology and many other social sciences has so far relied largely on the limited between-person methods, it remains unknown how many theories and conclusions will change once within-person methods are applied. Appendix B shows how two influential theories in the field of motivation seem likely to change substantially once within-person methods are used to examine their within-person statements and hypotheses. Since many psychological theories make assumptions about within-person patterns and developments, but have so far relied largely on between-person methods for the study of these assumptions, we currently find ourselves facing a widespread theory-method gap. This is a risk to the credibility of research findings. Although the debate about this problem is not new (e.g., Asendorpf, 1993; 2000; Kuhl, 1977; Lewin, 1930; Molenaar, 2004; Valsiner, 1986; Wottawa, 1981), the current time may be a crucial moment for new attempts to bring more appropriate methods to the research on theories about within-person patterns.

The threat to research’s trustworthiness that is brought about by the theory-method gap is met with a currently vibrant research on novel and re-discovered within-person methods (e.g., Reitzle & Dietrich, 2019; Beck & Jackson, 2021). The need for personalization in many research areas and applied areas puts further pressure on scientists and methodologists to provide appropriate solutions for the *description of individuals*. The debate about the limitations of between-person methods and about the unique contributions of within-person methods appears to see a renaissance since Molenaar’s (2004) manifesto on “Bringing the person back into scientific psychology” (see also Barbot et al., 2020, Renner et al., 2020). With these developments converging, this may finally be the moment for a proactive revision of psychological theories with the goal of reaching truly personalized descriptions, and in the long run, personalized treatments.

One justification that has often been brought up in defense of nomothetic, between-person methods is the goal of trying to find laws and rules of behavior that apply universally to everyone (e.g., Eysenck, 1954; Whitely, 1983). However, this article has summarized the reasons why between-person statistics may fail to describe even a single person in the studied sample, which implies that nomothetic methods do not necessarily describe universal rules or laws applying to everyone, or anyone at all. In addition, there is mounting evidence that many phenomena are more heterogeneous, less universal, than nomothetic approaches assume (e.g., Hoemann et al., 2017; see also Halvor Teigen, 2002).

Moreover, ever since the primary focus on nomothetic research topics was established in psychology, both research and real-life applications have moved forward. Although one-size-fits-all questions have resided in many fields, personalized approaches and studies of between-person heterogeneity have gained terrain. In education, personalized learning aspires to assess person-specific learning needs to provide each student with the tailored support that they currently need (e.g., Dumont, 2019). In personalized medicine – including personalized psychiatry – the patient’s individual needs are assessed for instance by sequencing their genome to identify individually matching treatments that would not necessarily work for other patients displaying similar symptoms (e.g., Jain, 2002; Senn, 2016; 2018). In personalized advertisement, people’s individual preferences are mapped to target them with advertisement fitting their individual personality and preferences (e.g., Zhu & Chang, 2016; Bang & Wojdynski, 2016). In real-life settings, large online sellers, such as Amazon or Netflix, employ individual predictions to target individuals with the offers of products that best fit their individual person characteristics. Personalization seems to be the theme of the hour both in business and retail as in many research areas.

In psychology, this represents a paradigm change away from nomothetic approaches towards more person-specific descriptions, predictions, and treatments. The demand for personalized solutions in applied fields, such as education or psychiatry, meets the supply of suitable methods for personalized predictions in many research fields, such as the pragmatic data science (e.g., Cho et al., 2002; Zhang, et al., 2013; Sarker et al., 2019 ), informatics (e.g., Jiang et al., 2013; Yin et al., 2010) and medicine (e.g., Senn, 2018; Zeevi et al., 2015). Considering that a clear demand is met with such a supply of personalized methods in other research fields, psychologists may want to start asking themselves if they want to leave such a core psychological topic – the description and prediction of human behavior, attitudes, preferences, and motivations – up to the applied fields while clinging onto a nomothetic paradigm limiting their scope to universal laws of behavior that appears increasingly unrealistic and of little use in many areas of life and research (see also Halvor Teigen, 2002; Salvatore & Valsiner, 2009).

This is not to say that nomothetic approaches should not be used. On the contrary, using within-person and idiographic methods does not rule out a nomothetic approach. However, based on the logical and empirical arguments summarized above, it seems a better strategy to treat generalizability and invariance of within-person findings across individuals as an empirical question itself that should be empirically tested and supported by evidence. This would mean that instead of assuming that nomothetic and between-person findings represent universal laws, we should test whether, how often, and under which circumstances within-person findings generalize across individuals before we report between-person trends expected to translate to individuals (for an example of such a bottom-up building of nomothetic insights, see the GIMME method; e.g., Beltz et al., 2016).

## 4. Do These Limitations of Common Between-Person Methods Imply That We Are at the Dawn of a New Credibility Crisis in Psychology?

The following main reasons suggest that a new credibility crisis might dawn upon psychological science: (1) the limitations and frequent misinterpretations of between-person methods that have been described here; (2) the widespread and often predominant use of these between-person methods in psychology; (3) the fact that these limitations have been criticized for many years by various authors in combination with the fact that these method critiques are often ignored in current psychological research (e.g., Molenaar, 2004; Simpson, 1951; Reitzle, 2013; Rogosa, 1980; Hamaker et al., 2015); (4) the fact that many people, including practitioners interested in personalized solutions, turn to psychology with questions about individuals that cannot be answered by the between-person methods that are applied to study these questions; (5) the fact that diverse within-person methods have been available for a long time, solve some of the limitations of within-person-methods, but are not yet embraced fully in many psychological research fields; and (6) the fact that within-person methods and personalized descriptions and predictions are not only needed but frequently used in applied fields that take a pragmatic data science approach and are interested in making trustworthy decisions about how to treat individuals, including banking (e.g., Galal et al., 2016; Hernández-Nieves et al., 2020;), advertisement (Zhu & Chang, 2016; Bang & Wojdynski, 2016), medicine (Senn, 2016; 2018), law enforcement (e.g., Tayebi et al., 2016), personalized content recommendation tailored to customer’s preferences in for example Amazon or Netflix (e.g., Gomez-Uribe & Hunt, 2015; Smith & Linden, 2017), and many more.

Together, these considerations imply, first, that we psychologists draw possibly false conclusions about our discipline’s main research object – persons; second, that we (could) have known about this fact for years; third, that we have chosen to not use the more appropriate and available methods, which would prevent us from drawing such false conclusions; and fourth, that other disciplines do this job better than us. Many psychological studies and entire research fields have chosen to turn a blind eye to the question whether and under which circumstances the predominant between-person methods properly describe the individuals in their samples and whether the nomothetic one-size-fits-all effects identified on the group level translate to the experiences of actual people. Many psychologists learn in their introductory statistics course that “Thou shall not draw conclusions about individuals based on group trends”, and then we spend the rest of our careers doing essentially just that.

Years and years of theoretical reasoning and empirical evidence show that the examination of within-person profiles and scatter plots can add to the examination of linear correlation or regression coefficients (e.g., Anscombe, 1973). Still researchers who employ profile analysis hear all the time from reviewers and colleagues that “someone still has to convince me that cluster analysis reveals anything that regressions do not” (personal experience of the author, many times repeated), and this despite of the fact that cluster and profile analysis is constantly used to great value as a standard data science tool in many disciplines and applied fields (e.g., de Oña, J., de Oña, & López, 2016; Perrotta & Williamson, 2018). It looks as though psychological science lags behind the insights that the more pragmatic use of data science offers to real-life decision making, possibly because psychologists tend to be committed to the a priori epistemological idea that a nomothetic approach should always take priority over the idiographic idea that different individuals may function in different ways. This epistemological belief has been challenged by numerous empirical findings, which show that much heterogeneity exists and how insightful it is to describe such heterogeneity (e.g., Kievit et al, 2013), as well as by many theoretical and epistemological arguments (e.g., Jacomy, 2020; Molenaar, 2004; Salvatore & Valsiner, 2009; 2010), novel integrations of idiographic with nomothetic approaches (e.g., Beltz et al., 2016), and by the fact that real-life practitioners who are interested in making efficient, trustworthy decisions and predictions about individuals seem to fare pretty well with their pragmatic use of both within- and between-person methods (e.g., DeMatteo et al., 2010).

Together, these considerations suggest that psychology may stand at the dawn of a new credibility crisis similar to the one we faced during the replicability crisis (e.g., Anvari & Lakens, 2018; Pashler & Wagenmakers, 2012) – a credibility crisis due to the fact that between-person methods and nomothetic theories fall short of the prediction of individual human behavior that real-life practitioners achieve by using pragmatic data science. Whereas the replicability debate addressed the problem that research findings may not be as invariant across samples and contexts as expected of them, the imminent next credibility crisis may reckon that *we do not even understand properly what happens in the original sample at hand*. In conclusion, the method critiques summarized here imply that psychological studies attempt to describe laws of behavior that are assumed to apply to everyone in the sample (nomothetic approach) and that actually may fail to describe many, or even all, persons in the sample. By doing that, psychology misses out on the understanding of what it should be most interested in: individuals (Barbot et al., 2020; Carlson, 1971; Molenaar, 2004). If we want to find out how groups function, we can ask sociologists. When we ask psychologists a question, we expect that their answer will help us understand what goes on *inside of people’s minds and lives*, and the current use and predominance of between-person methods lets us miss exactly these points.

## 5. Pathways Towards a Pro-Active Reckoning in Psychological Science

The theoretical and methodological arguments that have been presented here may give reasons to worry about the question whether we know what we think we know about what goes on in people’s minds and lives. Systematic empirical research is needed to find out how many studies in various fields of psychology may be affected by the possible misinterpretations of results achieved with between-person methods being interpreted as if they described within-person patterns.

In order to avert a loss of credibility similar to the one that followed from the previous replicability crisis (Anvari & Lakens, 2018; Pashler & Wagenmakers, 2012), psychologists may want to strive for a proactive reckoning by exploring the prevalence of the various possible misinterpretations of between-person methods in their field, by exploring the possibility of complementing their usual methods with a larger variety of within-person methods, and by adapting their theories. Further theory development could strive to adapt theories to the fact that most of them have so far been examined mainly with between-person methods and should therefore limit their statements to the conclusions that can be drawn based on such methods. In addition, or alternatively, further theory development could strive towards extending nomothetic theories to include reflections on possible heterogeneity in within-person patterns, by making sure that within-person assumptions are systematically tested with within-person methods.

The following research questions could serve as directions for a pro-active reckoning about the current limitations of psychological methods, for the purpose of first understanding and then closing the current theory-method gap:

(RQ1) How many studies in different fields of psychology show misinterpretations of between-person methods in the form of results obtained with between-person methods being interpreted as if they described within-person patterns^6^ without providing the required evidence that such an intra-individual interpretation is appropriate?

(RQ2) How many studies in different fields of psychology consider the known limitations of between-person methods that have been described in this article, such as heterogeneity hiding behind overall trends (as described in Simpson, 1951; Anscombe 1973; Matejka & Fitzmaurice, 2017), or different individuals driving different paths in structural equation models (Reitzle, 2013) when they use methods for which these considerations are relevant?

(RQ3) Which psychological theories/conclusions will have to change substantially due to contributions by within-person methods, and in what ways do the conclusions have to change? What exactly are the unique insights provided by within-person methods that go beyond those provided by between-person methods in psychological theory development? For some first considerations on this question, see Appendix B.

(RQ4) What are the current limitations of available within-person methods, and what are the solutions to these limitations? For some first considerations on this question, see Appendix A.

(RQ5) What unique contributions can between-person and within-person methods, respectively, make to applied fields aiming at personalized diagnostics and treatments, as for example personalized medicine?

The empirical reckoning of psychology with the goal to overcome possible theory-method gaps could apply for instance the following methods to examine the research questions suggested above:

*Reviews* could re-examine the published research to find out how frequently between-person methods are used to examine within-person assumptions, or how often known limitations of between-person methods are (not) reflected in the analysis and interpretation of between-person methods.

*Surveys* could ask psychological researchers across various research topics and subdisciplines about (1) their awareness about limitations of between-person methods, available within-person methods, and possible theory-method gaps in their field, and (2) the frequency of between-versus within-person methods being applied in their psychological studies.

*Revisiting established theories:* Theories that have been addressing within-person assumptions but have so far been supported mainly by between-person methods could be revisited by adopting appropriate within-person methods suited to test their within-person assumptions (for suggestions regarding specific theories, see Appendix B). This could be done either by conducting new studies collecting new data, or by using existing datasets associated with well-established theories that have previously been examined mainly with between-person methods and re-analyzing them in secondary data analyses with within-person methods.

If taking this proactive approach of a systematic reckoning to avert a possible new credibility crisis, we can build upon the lessons learned from the previous replicability crisis. The replicability debate can teach us that it may be wiser to acknowledge systematic problems in our use of research methods rather than sweeping them under the carpet by ignoring or downplaying the warning voices. We can also keep using the power of crowdsourcing for the systematic empirical revisiting of established theories, for instance by adopting a many-analysts approach (e.g., Aczel et al., 2021; Bastiaansen et al., 2020; Silberzahn et al., 2018) on open data to answer the question whether and in what cases a re-analysis of data used in previous studies supports the published conclusions or contributes new insights. Panel discussions could be organized to explore the scope of the problem, its implications, and available solutions. Open data could speed up the systematic re-analysis of data on established, well-studied theories. The experience of having faced the replicability crisis together as a field and having grown from it, can teach us to keep in mind that research always changes and hopefully increases in its wisdom and that such changes, even when affecting one’s own much-loved research field, do not have to be perceived as a threat, nor as a failure, but can be welcomed as a form of insight and growth. In sum, the flourishing research on within-person methods promises to enrich the psychological methods portfolio in likely game-changing and highly relevant and timely ways. Embracing and discussing the novel insights it contributes, along with their methodological, theoretical, and epistemological implications (and their own limitations), promises to be a valuable and important task for psychological science.

## 6. Getting Ready for the Future by Learning from the Past

It should be noted that this article is far from being the first to point out a mismatch between the within-person theories being studied with between-person methods. That this mismatch has led psychological research into a validity crisis has been pointed out before by Lundh (2019). The same author also already pointed out that the currently trending attempts to personalize medicine are particularly in need of person-oriented (within-person) methods, a thought echoed by Lundh & Falkenström (2019) and also mentioned by Wiedermann et al. (2016). The calls for more within-person methods and notes of concern about the theory-method mismatch addressed in this article go back many decades to early works by, for instance, Allport (1961; 1962), Carlson (1971), Stern (1911), Magnusson (1988; 1999), Cattell et al. (1947) and Windelband (1894/1998). The various historical roots and decades-old but much-needed solutions of idiographic and within-person methods have been summarized in the early issues of the Journal for Person-Oriented Research (see e.g., Lundh, 2015; Valsiner, 2015; Bergman & Lundh, 2015; Valsiner, 2016).

This article aspired to build upon but reach beyond these previous considerations by pointing out the extent to which ignoring the long-known theory-method mismatch has brought psychological science to the brink of a new credibility crisis and by linking the addressed problems to an overview of available solutions and a research program aimed at helping psychological science meet the demands of the currently trending personalized diagnostics and intervention.

## 7. Further Theory and Method Development Needed to Better Describe and Predict Individuals

It should also be noted that this article’s list of limitations of between-person methods with regard to the aspects about individuals that these methods fail to describe is far from complete. For instance, while this article has emphasized the theory-method gap with regard to dangers of using standard group-oriented *analyses* to draw conclusions about laws applying to individuals, other authors have pointed out that already the previous step of *measurement* has to be improved in order to avoid a further loss in the credibility of psychology and the social sciences. Please see for instance Flake and Eid (2021) for a general discussion of prevalent measurement issues in psychology; and Bergman (2017) for a discussion of changes to measurement practices that would be needed to obtain more trustworthy measures for the assessment of individuals.

Likewise, although the list of within-person methods that has been presented here may help solving some of the listed limitations of between-person methods, it is far from complete. Particularly promising avenues are current developments building on dynamical systems theories, such as nonlinear dynamic systems and nonstationary dynamical systems approaches (e.g., Molenaar et al., 2016). While the main aim of this article was to point out that there are many widely studied within-person methods available to overcome the listed limitations in between-person methods, there is a rapid development of novel and groundbreaking within-person methods reaching beyond the solutions presented in this article.

Finally, there will be more to do, even if future studies set out to avoid the here-described theory-method gaps by making sure that theories about within-person patterns are examined with appropriate within-person methods. Many psychological theories only make statements about between-person differences, possibly in part due to the fact that the available between-person methods may have shaped and limited the ways we dare to think about psychological processes. On the other hand, many of the studies that use within-person methods (e.g., cluster analyses) or idiographic methods present their approaches as exploratory and not theory- but data-driven. One challenge for further research on individuals will be to close this gap by developing theories and theory-driven hypotheses that explicitly address within-person patterns and processes and by linking within-person methods more strongly to such theories and theory-driven hypotheses (see also the recent debate about the theory crisis and possible solutions, e.g., Eronen & Bringmann, 2021; Fiedler, 2017; Fried, 2020; Guest & Martin, 2021; Haslbeck et al. (in press); Muthukrishna & Henrich, 2019; Meehl, 1967; 1990; Oberauer & Lewandowsky, 2019; Smaldino, 2019; Vaidyanathan et al., 2015). It also seems that the available confirmatory hypotheses-testing features of within-person analyses (e.g., confirmatory latent profile analysis or confirmatory network analysis) are underused and widely unknown. And finally, there is a long way between describing or predicting individuals and finding the right treatment for the right person at the right time. We have only started this long journey. Let’s take a first step by applying within- and between-person methods mindfully so as to match theories and hypotheses.

## Figures and Tables

**Figure 5:**
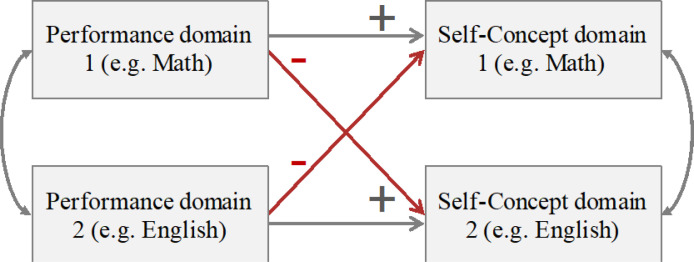
Assumed relationships between performances and self-concepts in different school subjects in dimensional comparison theory.

## Footnotes

1 Simpson’s paradox: An effect (e.g., a positive correlation) which ap-pears within groups of observations (e.g., groups of multiple measure-ments per person) but disappears when these separate groups are com-bined and the effect is recalculated without taking the former grouping into account. For instance, a correlation coefficient being negative within individuals (across repeated measures) but becoming zero or positive once the nestedness of repeated measures in individuals is ignored.

2 Definition of ergodicity: Ergodicity in a dynamical system exists if the average behavior of the system (e.g., a sample of individuals) can be de-duced from the behavior of individual measurement points (e.g., one indi-vidual). For instance, ergodicity would be given if the developmental trajectory of one given person over time is a good indicator for the average development of the system over time. Lack of ergodicity implies a hetero-geneity between the individual measurement points (in this example: individuals) in the sense that one given individual’s trajectory is not nec-essarily a good estimate for how we can expect the system to develop.

3 Please note: Such multilevel models on repeated measures nested in individuals constrain the model to be invariant across cases (individuals), and typically report only one within-person coefficient for the entire sam-ple, in line with this assumption of homogeneity of such coefficients across individuals. This may not be adequate if ergodicity is lacking (if for instance the within-person correlation coefficient differs between individ-uals or if the in-the-moment profiles of the studied variables differ be-tween situations and their prevalence differs between individuals).

4 Please note: Such multilevel models on repeated measures nested in individuals constrain the model to be invariant across cases (individuals), and typically report only one within-person coefficient for the entire sam-ple, in line with this assumption of homogeneity of such coefficients across individuals. This may not be adequate if ergodicity is lacking (if e.g., the within-person correlation coefficient differs between individuals or if the in-the-moment profiles of the studied variables differ between situations and their prevalence differs between individuals).

5 That is, assuming that the cut-off that distinguishes between item en-dorsement and item rejection is located at the middle of the response scales for both variables, otherwise the area representing co-endorsement is not necessarily equivalent to a quadrant in the scatter plot.

6 Within-person patterns being defined as a) the covariance among two or more variables, b) the profile (or co-affirmation / co-negation) of more than one variable, c) the change in one (or more) variable(s) over multiple time points

## Action editor

Lars-Gunnar Lundh served as action editor for this article.

## Appendix A: Limitations in the currently available within-person methods

Not all of the above-mentioned limitations of between-person methods with regard to their capabilities of describing individuals are solved by the available within-person methods. *Essentially, within-person methods just bring the problems concerning between-person analyses mentioned above to the next more specific level concerning differences between multiple measurement time points*.

*Example 1*: Where between-person methods fail to describe heterogeneity between individuals (see above section 1.3), within-person methods may fail to describe heterogeneity between situations, within individuals. If the heterogeneity between situations comes in the form of non-stationary effects (e.g., a cross-sectional or lagged correlation coefficient or a lagged auto-regression changing over time), then certain time series models capable of revealing non-stationarity can be a solution (see e.g., Molenaar et al., 2016). However, if the heterogeneity between situations comes in the form of distinct state profiles that may differ between the various situations that an individual experiences (e.g., profile 1: interesting, easy but emotionally taxing situations; profile 2: uninteresting, easy and emotionally taxing situations; profile 3: interesting, difficult and emotionally taxing situations, profile 4: interesting easy but not emotionally taxing situations, etc.), then correlation-based methods may not reveal the full scope of such within-person heterogeneity because the heterogeneous profiles may hide behind the person-specific and time-specific correlation coefficients.

*Example 2:* Where the analysis of intra-individual profiles or co-occurrences solves the problem that unexpected groups of individuals with distinct within-person patterns can hide behind overall trends, these within-person profile and co-occurrence network analyses may overlook that within each person, there may be groups of situations with profiles or co-occurrence patterns defying the cross-situational trend seen in the same individuals. Dynamic models for non-stationary processes can accommodate for covariance patterns differing between time points or time periods within a person, but they cannot solve the problem that distinct state profiles may hide behind such correlation trends.

*Example 3:* Where multilevel analyses decomposing the within- and between-person (co-)variance help to detect Simpson’s paradox by showing differences between the covariance patterns of the within-person level and the between-person level (please see sections 1.1; 2.1; and 1.2; 2.2 above, as well as Brose et al., 2020; Völkle et al., 2014), they may overlook Simpson’s paradox with regard to differences in covariance patterns between other groups of situations (please see below).

### A.1 Limitations with regard to understanding heterogeneity within individuals

While the within-person methods described in this article may help to describe *heterogeneity between individuals* in regard to within-person patterns, they are limited in regard to their capacity to describe *heterogeneity within individuals between situations*. Heterogeneity does not only exist between individuals in regard to their within-person trajectories, covariance patterns, profiles or co-occurrences. *Heterogeneity can also exist within a person, in the way that situation-specific profiles of variables may differ from one moment to the next*.

For example, within a person, different situations may differ with regard to (1) the in-the-moment profiles (see e.g., Dietrich et al, 2019), or (2) the co-occurrence pattern among repeatedly measured variables. The first of these problems can be addressed by analyzing in-the-moment profiles, for instance with multilevel latent profile analyses, see Dietrich et al., 2019, or the so-called ISOA approach^7^ (Bergman et al., 2012), whereas the second problem can be addressed by analyzing in-the-moment co-occurrence patterns (Moeller et al., 2018a).

Third, there may be unexpected bi-variate distributions hiding behind within-person correlations or regression coefficients of repeatedly measured variables (as described by Anscombe, 1973 and Matejka, & Fitzmaurice, 2017), which implies that a within-person correlation or regression does not imply that the studied variables show the relation that we expect based on their within-person correlation or regression coefficient. Scatterplots, which show the bivariate distributions of the repeated measures for each person, may help to rule out such unexpected bivariate distributions hiding behind within-person trends.

Fourth, the problem of Simpson’s paradox or lack of ergodicity is not entirely and automatically solved by the variance decomposition into within- and between-person variation that is often hailed as its solution, because of the following reasons: It is possible that within a person there are groups of situations in which a regression or correlation coefficient looks different than the overall coefficient found in the same person. For example, in an intensive longitudinal study with repeated surveys per person, the relation between two emotions could be negative within each school lesson, but might look positive for the same person if we only examine the within-person versus between-person covariance and instead of the here more appropriate decomposition into (1) the within-lesson – within-person variance, (2) the within-person – across lesson variance, and (3) the between-person variance. Some readers may argue that this can be easily solved by taking additional layers or clusters of observation into account by adding a third level to the multilevel analyses that are often used to address lacking ergodicity (e.g., Brose et al., 2020; Völkle et al., 2014), but there is a crucial limitation to that option: *We cannot make sure to know all the relevant boundary conditions that may influence a within-person regression or correlation coefficient*, and in intensive longitudinal data, there are typically many possible – often unknown – contextual sources of variation, combined with the expectation that the studied phenomena are highly time- and context-sensitive (hence the need to study them with intensive longitudinal methods). If fluctuation across contexts and time is expected in intensive longitudinal studies, and we keep in mind that many studies and findings in research with intensive longitudinal studies are rather new and often the first of their kind, we have strong reasons to believe that there is much that we do not know about the context- and time-related sources of variation in intensive longitudinal studies. Consequently, we should assume that within-person correlation or regression coefficients may depend on certain characteristic of the contexts and times in which the data were collected. This in turn implies that Simpson’s paradox or a lack of ergodicity can exist without us being able to detect it, for instance a within-person regression or correlation coefficient is positive within the units of an unknown/unexplored boundary condition (e.g., within school classes, within days, within certain contexts such as school, home, leisure, or within each interaction with a person’s significant other), while the same within-person regression or correlation coefficient can be negative for the same person across these units of an unknown/unexplored boundary condition. While there are some tutorials helping to detect and address Simpson’s paradox in longitudinal data (e.g., Kievit et al., 2013), we can expect the problem of unknown boundary conditions to limit the trustworthiness of coefficients concerning incompletely understood phenomena that are expected to fluctuate strongly between contexts and time points.

### A.2 The question of who walks which paths (Reitzle, 2013) remains unanswered in within-person co-occurrence analyses

A limitation of the co-occurrence network is that it currently does not distinguish between person-specific (idiographic) networks and between-person (nomothetic) networks, as some of the co-variance-based network approaches do (e.g., Beltz et al., 2016; Beck & Jackson, 2020). While each bivariate edge (line between two variables) can be interpreted as the number of times these variables were co-endorsed or co-occurred within the same person^8^, we do not know whether the same individuals^9^ account for the co-occurrences represented by the other edges (lines) in the same network. Thus, we currently do not know “who walks which path” (see Reitzle, 2013 and section 1.5 above), nor do we know the percentage of individuals showing intra-individual patterns in line with the *entire* inter-individual co-occurrence network, as proposed by Asendorpf (2000). To solve this, we need to examine person-specific co-endorsement networks.

## Appendix B: What Psychological theories can we expect to change when we start using more within-person methods?

It may seem surprising how many established, much-cited psychological theories still predominantly use between-person methods to study within-person patterns, considering the many years of debates about the limitations of between-person methods (e.g., Asendorpf,

2000) and the number of available within-person approaches (e.g., Barbot et al., 2020; Fleeson, 2004; Molenaar, 2004; Renner et al., 2020; Reitzle & Dietrich, 2019; Beck & Jackson, 2021). Some research areas have widely adopted some (but usually not all) within-person methods described above. For instance, a number of studies in the area of personality psychology, clinical psychology, and emotion research examine within-person variance with multi-level models and within-person network models (e.g., Beck & Jackson, 2019; Haslbeck & Ryan, 2020; Hoffart & Johnson, 2020). In contrast, in some fields of motivational psychology, research on education, and in research related to problems of personalized learning, there may still be a need for within-person approaches to complement the currently widely prevailing between-person approaches. In the following, I introduce research areas that currently use mostly between-person methods to study within-person patterns. All subsequently addressed theories are relevant for the improvement of personalized learning that this article has addressed. Please note that these theories are mentioned as exchangeable examples or placeholders for many other theories relying on mostly between-person methods, in order to make the point that future studies may want to make more use of the available within-person methods. The following discussion is not meant as a critique but rather as a proposal for new directions in further studies on these important, plausible, and well-studied theories, because of the importance, plausibility, and popularity of these theories, which can serve as role-models for the further development and paradigmatic changes in other, not subsequently mentioned, theories.

### Dimensional Comparison Theory (DCT)

Dimensional comparison theory (DCT) is a much-cited theory in education (e.g., Möller & Marsh, 2013; Möller et al., 2016). It states that students compare within themselves how they perform in one school subject (e.g., Math) with how they perform in another (e.g., English). A central conclusion in the literature on the DCT is that “Students performing better in the math than in the verbal domain tend to have lower self-perceptions of their verbal ability than do students with identical verbal ability but lower math ability (and vice versa)” (Möller & Marsh, 2013, p. 544).

Although DCT at its core draws conclusions about within-person comparisons of performances and self-concepts within and across school subjects, the large amount of studies on DCT almost exclusively rely on between-person analyses of covariance among these variables (but see also the less frequent experimental and longitudinal studies on DCT, e.g., Helm et al., 2016; Möller & Husemann 2006; Niepel et al., 2021; Strickhouser & Zell, 2015). The prevalence of between-person methods in DCT research implies that there is a theory-method gap in many previous studies on DCT, and that research on DCT risks being affected by the limitations of between-person methods in describing individuals that were described in the sections 1.1 to 1.5 of this article). In particular, research on the DCT may or may not be affected by the problems of heterogeneity hiding behind overall regression coefficients (see section 1.3 and Anscombe, 1973), the problem that separate groups of individuals may drive the covariance in different paths in the model (section 1.5 and Reitzle, 2013), and the problem that the examined covariance does not carry any information about the relations in raw scores (section 1.4 and Moeller et al., 2018a). Thus, based on the prevalence of between-person methods that have been used to study the DCT, we do not know whether and for whom the self-concept in one subject is “higher” than the self-concept in another, we do not know how much heterogeneity may hide behind any one path in the model, and we do not know how many individuals are described properly by one or by multiple paths of the model. Therefore, it is unknown whether the conclusions of the much-cited DCT will be confirmed by within-person analyses. There are first studies assessing within-person variation in DCT-related measures with experimental, diary and experience sampling method approaches (Helm et al., 2016; Möller & Husemann 2006; Niepel et al., 2021; Strickhouser & Zell, 2015). These and further within-person studies on the hypothesized within-person relations among performances and competence self-concepts in different school subjects are needed to find out whether the hypothesized within-person comparisons can be found within persons.

These questions matter, because DCT is a very influential theory about students’ motivation. Its findings and their implications are crucial for the understanding of how students can be motivated, which in turn is crucial for the understanding of how personalized learning can address individual students’ motivation. That makes it necessary to test the statements of the DCT with the appropriate methods. DCT is so popular and known that it is a beacon with signaling effects. Demonstrating that within-person methods contribute unique insights to such a widely-studied subject is likely to inspire researchers working on other theories to revisit theirs.

### The Dual Model of Passion

The Dual Model of Passion is another extremely influential theory about motivation. It states that passion is a form of motivation that consists of a person liking an activity, finding that activity important, investing time and energy with that activity, identifying with the activity, and calling the activity their passion (e.g., Vallerand et al., 2003). The Dual Model of Passion distinguishes between a positive, desirable form of passion called harmonious passion (HP) and a harmful, undesirable form of passion, called obsessive passion (OP). Both forms of passion are measured with different scales (Vallerand et al, 2003). While HP is correlated with and predicts all sorts of positive outcomes, such as positive emotions, indicators of wellbeing, and achievement, OP is correlated with and predicts all sorts of negative outcomes, including negative emotions, the risk of getting injured, burnout-symptoms, and many other undesirable experiences (for overviews, see e.g., Vallerand, 2010; Vallerand & Houlfort, 2019). The vast majority of studies on the Dual Model of Passion employ between-person methods, mostly the analyses of between-person correlations or regression coefficients, often in the context of structural equation models. Nevertheless, one frequently finds that these studies are interpreted as if they described within-person patterns of the passion forms HP, OP, and their respective outcomes, for instance in sentences such as “people with a harmonious passion should be able to fully focus on the task at hand and experience positive outcomes” (Vallerand, 2012, p. 4) and “people with an obsessive passion can thus find themselves in the position of experiencing an uncontrollable urge” (Vallerand, 2012, p. 3). Even some studies that explicitly set out to examine within-person combinations of harmonious and obsessive passion use between-person methods (mostly analyzing between-person covariance; Schellenberg et al., 2019). This demonstrates that there are many different layers and aspects to within-person variation that are often hard to address in one single study, leaving room for future studies on further aspects of within-person patterns of HP and OP (e.g., co-occurrence analyses or profile/cluster analyses).

A frequent statement posits that passionate individuals experience either a predominant HP or a predominant OP, and could thus be classified into “mainly harmonious individuals” and the “mainly obsessive individuals” (Philippe et al., 2009). However, the few within-person studies on within-person profiles of HP and OP revealed that there were almost no individuals with higher obsessive than harmonious passion, and that most individuals experienced HP and OP together in either both high, or both moderate, or both low levels (e.g., Moeller et al., 2015; Wang et al., 2008). This suggests that within-person methods can really change our interpretations of the experiences that individuals make and that further within-person studies on the Dual Model of Passion promise to make interesting contributions to our understanding of the ambivalent motivation experienced by individuals.

The insights gained by discussing these two examples of the DCT and the Dual Model of Passion are likely to transfer to many other psychological theories. The goal of this discussion was not to call out specific theories, but to describe these insights as an inspiration to demonstrate how much more insight and what tremendous paradigmatic changes may be gained from integrating the available within-person and between-person methods more systematically.
